# Development and immunobiological evaluation of nanoparticles containing an immunodominant epitope of herpes simplex virus

**DOI:** 10.1049/nbt2.12043

**Published:** 2021-03-30

**Authors:** Gabriel M. Hilario, Fernando B. Sulczewski, Raquel Liszbinski, Larissa D. Mello, Gustavo Hagen, Tiago Fazolo, Jayme Neto, Eliane Dallegrave, Pedro Romão, Tanira Aguirre, Luiz C. Rodrigues Junior

**Affiliations:** ^1^ Laboratorio de Imunovirologia Universidade Federal de Ciências da Saúde de Porto Alegre Brazil; ^2^ Laboratório de Nanotecnologia Universidade Franciscana Brazil; ^3^ Laboratório de Imunologia Celular e Molecular Universidade Federal de Ciências da Saúde de Porto Alegre Brazil; ^4^ Laboratório de Pesquisa em Toxicologia Universidade Federal de Ciências da Saúde de Porto Alegre Brazil; ^5^ Laboratório de Imunoterapia Universidade Federal de Ciências da Saúde de Porto Alegre Brazil

## Abstract

*Herpes simplex* virus (HSV) 1 and 2 are viruses that infect individuals worldwide and for which there is no cure or vaccine available. The protective response against herpes is mostly mediated by CD8 T lymphocytes that respond to the immunodominant SSIEFARL epitope. However, there are some obstacles concerning the use of free SSIEFARL for vaccine or immunotherapy. The aim of this study was to evaluate the feasibility of nanoencapsulation of SSIEFARL and its immunostimulatory properties. Nano/SSIEFARL was produced by interfacial polymerization in methylmetacrylate, and the physico‐chemical properties, morphology and immunobiological parameters were evaluated. To evaluate the ex vivo capacity of Nano/SSIEFARL, we used splenocytes from HSV‐1‐infected mice to enhance the frequency of SSIEFARL‐specific CD8 T lymphocytes. The results indicate that Nano/SSIEFARL has a spherical shape, an average diameter of 352 ± 22 nm, the PDI was 0.361 ± 0.009 and is negatively charged (−26.30 ± 35). The stability at 4°C was 28 days. Also, Nano/SSIEFARL is not toxic for cells at low concentrations in vitro and it is taken up by JAWS II dendritic cells. No histopathological changes were observed in kidneys, liver and lymph nodes of animals treated with Nano/SSIEFARL. Nan/SSIEFARL increased the production of IL‐1β, TNF‐α and IL‐12 by the dendritic cells. Finally, Nano/SSIEFARL expanded the frequency of SSIEFARL‐specific CD8+T lymphocytes at the same rate as free SSIEFARL. In conclusion all data together indicate that SSIEFARL is suitable for nanoencapsulation, and the system produced presents some immunoadjuvant properties that can be used to improve the immune response against herpes.

## 1 INTRODUCTION

*Herpes simplex* viruses (HSVs) are dermatopic/mucosal viruses that cause skin lesions and establish latency in the nervous ganglion closest to the site of infection. It represents a significant public health burden, and there is no cure or available vaccine [1, 2]. The HSV‐1 often infects the orofacial region and HSV‐2 the genital region. After the first viral cycle in epithelial cells, the virus accesses local sensory nerve, where it establishes latent infection [3]. HSV also infects dendritic cells via the DC‐SIGN receptor, in these cells it impairs the cell activation, which is characterized by a reduction in the expression of MHC II, MHC I and CD86 and in the production of IL‐12 [4, 5, 6]. When the virus is reactivated from latency, it returns to the same mucosal site of primary infection. The frequency of recurrences varies from individual to individual, but some of them may have up to 10 recurrences annually [7, 8, 9].

The virus latency is controlled by helper T (CD4) and cytotoxic T (CD8) lymphocytes that remain juxtaposed to the nerve sheath sending factors to promote viral latency [10]. CD8 T lymphocytes are also found in the local mucosal tissue and after each HSV recurrence the number of remaining resident cells possible determine the time and intensity of next reactivation [11, 12]. In this dynamic the majority of CD8T lymphocytes are specific to peptide SSIEFRAL, that is an immunodominant epitope located in glycoprotein B from HSV‐1 and HSV‐2 [13, 14]. After one single episode of HSV‐1infection, in the mouse model, over 80% of CD8 T lymphocytes are directed against SSIEFARL [13, 15]. Thus, SSIEFARL is an important candidate for application in HSV‐1 and HSV‐2 vaccination or immunotherapy. However, there are obstacles to use SSIEFARL in its free form, especially for mucosal or dermal application. Peptides are degraded by peptidases and quickly removed from the site of application, leading to a low rate of phagocytosis and insufficient immune response [16, 17].

Over the last decades, several different systems to improve the delivery of viral peptides for immunological stimulation have been developed, such as liposomes, virosomes, immune stimulation complexes (ISCOMs) and polymeric nanoparticles (PNs) [18]. Among these systems, (PNs) are suitable for encapsulation of hydrophilic peptides, while they are biodegradable, present long time of peptide release and can present adjuvant properties [19, 20, 21]. PNs produced with an aqueous core can efficiently entrap and protect hydrophilic oligonucleotides and peptides from degradation or interaction with tissue/serum proteins [22, 23, 24]. The encapsulation of antigenic peptides also improves the time of retention in the site of application, which increases the rate of phagocytosis and the delivery to local lymph nodes and activation of specific T and B lymphocytes [25, 26]. PNs are promising systems to overcome some limitations of using peptides in immunotherapy. Thus, the aim of this study was to encapsulate the SSIEFARL peptide into PNs and to evaluate the adjuvant properties of the system for future applications in dermal/mucosa immunotherapy against HSV‐1 and HSV‐2 recurrence. To our knowledge, this is the first study reporting the development of a PN containing the HSV immunogenic SSIEFARL peptide.

## 2 MATERIALS AND METHODS

### 2.1 Nanoparticle preparation and SSIEFARL encapsulation

The preparation of PNs was performed according to the method adapted from Lambert et al. [27]. Briefly, a lipophilic phase composed of 8 g sorbitan monoleate (Span^®^ 80, Millipore) and 1.5 g caprylic and capryic acid triglycerides was added under magnetic stirring to a hydrophilic phase composed of 200 µl absolute ethanol and 800 µL deionized water. Simultaneously 10 µL of methyl methacrylate cross polymer (MMA) was added. The volume of ethanol and the temperature of preparation were experimentally adapted. The emulsion remained under magnetic stirring at 500 rpm for 4 h. After complete polymerization, the emulsion was washed with deionized water (1:1), the final volume was approximately 15 mL, which was centrifuged at 14.500 rpm over 45 min. The oil phase and the interface were removed and the pellet containing the PNs was washed two more times. To produce PNs containing the HSV peptide, 60 µL of a solution containing 50 µg/ml of SSIEFARL was added to the aqueous phase making the theoretical final concentration of 200 ng/ml of SSIEFARL. The product was PNs dispersed in aqueous medium (empty) and when SSIEFARL was added it was named Nano/SSIEFARL. To optimize the production, four different strategies for preparation were tested, two in different water temperatures (40°C or 90°C), and other two either using or not using ethanol.

### 2.2 Particle size distribution, polydispersity index and zeta potential analysis

Particle size (z‐average hydrodynamic diameter) and polydispersity index (PDI) of PNs were determined by dynamic light scattering (DLS) (Zetasizer^®^ Nano ZS90, Malvern Instruments Ltd) at a fixed angle of 90°. Each formulation was diluted in ultrapure water (100×) and analysis were carried out at 25°C. The zeta potential values were determined by the electrophoretic mobility after dilution of PNs in 10 mM NaCl aqueous solution (500×). Both diluents were previously filtered (0.45 µm, Millipore^®^).

Empty PNs and Nano/SSIEFARL were kept under refrigeration at 4°C for a period of 28 days. During the incubation period, aliquots were removed weekly and particle size, PDI and zeta potential were evaluated and data were compared to the day of PNs preparation (day 0).

### 2.3 Morphological analysis of Nano/SSIEFARL

The morphological characteristics of produced PNs were analysed by transmission electron microscopy (TEM) using the microscopy JEOL 12,000 ExII in 120 Kv. Briefly, the Nano/SSIEFARL were diluted in ultrapure water 1:100 (v/v) and set in grids covered with Form‐Carbon film and support of 300 mesh. Uranyl acetate 2% (v/v) (Agar Scientific. ESSEX) was used as negative contrast. Also, unstained blank PNs were used as experimental negative control.

### 2.4 Loading, encapsulation efficiency and peptide release

The loading of peptide incorporated into PNs was analysed by confocal laser scanning microscopy (CLSM) and quantified using a high‐performance liquid chromatography method (HPLC) with fluorometric detection. To perform the confocal microscopy, the Nano/SSIEFARL was produced with the peptide conjugated to 6‐FAM (6‐carboxifluorescein) and the fluorescence was analysed in Olympus FV1000 confocal microscope. Unstained blank PNs were used as experimental negative controls. For HPLC quantification, Nano/SSIEFARL were prepared as described in 2.1, however SSIEFARL conjugated to fluorescein (FITC) was encapsulated. Samples were diluted in acetonitrile:water (4:1), as indicated by the peptide supplier, sonicated for 15 min, filtered (0.45 μm) and assayed. The chromatographic system consisted of a C18 column (150 mm × 4.6 mm, 5 μm particle size, 110 Å pore diameter, Phenomenex Gemini) and HPLC equipment (Shimadzu, Kyoto, Japan) with a LC‐20AT pump, a RF detector, and a SIL‐20A auto‐sampler. The data were acquired with Shimadzu CLASS‐VP software. The mobile phase composition was acetonitrile and 0.2 M potassium phosphate bufferplus0.05% (v/v) trifluoroacetic acid at pH 6.7 at a ratio of 20:80 (v/v) pumped at isocratic flow rate (0.7 mL/ min^−1^). The injection volume was 20 µL and column temperature was controlled at 35°C. Fluorescence detection was performed with excitation and emission wavelengths at 490 and 520 nm, respectively. The method was linear in the range of 0.105 to 10.5 ng/mL, precise over 6 h, accurate, and specific. To evaluate the peptide release, we used microcentrifuge tubes with the dialysis membrane attached to the opening, five tubes were used considering one tube for each time point. All tubes were filled with phosphate buffered saline (PBS) pH 7.2 and the membrane of each one was filled with 50 µL (4.55 × 10^8^ PNs) of Nano/SSIEFARL‐FITC. All membranes were in contact with PBS. The tubes were kept under agitation at the speed of 20 rpm in an orbital shaker, before starting the rotation, the PBS from tube time zero was removed and subsequently the PBS from tube 1, 3, 9 and 24 h. The fluorescence was quantified at with excitation and emission wavelengths at 490 and 520 nm, respectively. Samples were analysed in a 96‐well black microplate in a SpectraMax M2e (Molecular Devices).

### 2.5 Calculation of the number of PNs by sample

The number of PNs present by volume of the produced suspension was calculated using the protocol described by Polleto et al. [28]. Briefly, three independent batches of PNs were produced and analysed by UV‐VIS spectrophotometry to determine the turbidity (*Ƭ*). The *Ƭ* (cm^−1^) was calculated using the absorption of each batch applied to the following Equations.

### 2.6 Cytotoxicity of PNs towards different cells in vitro

The toxicity of PNs was initially determined in JAWS II cell line (ATCC CDL‐11,904). JAWS II were cultured in Alpha MEN Media with ribonucleotides and deoxyribonucleotides (Sigma), supplemented with 20% fetal bovine serum (FBS) (Sigma) and 5 ng/mLof murine GM‐CSF (Peptrotech). Cells were seed in 96 well plate and cultured in incubator with 5% of CO_2_ atmosphere. When cells reached 70% confluence the medium was replaced by another with the same composition adding 9,1 × 10^9^, 1,8 × 10^10^or 4,5 × 10^10^ PNs/mL of Nano/SSIEFARL. Cells cultured only with media, free SSIEFARL (0.3 µg/mL) and empty PNs (1.8 × 10^10^ PNs/mL) were used as controls. At 24, 48, 72 or 96 h after incubation with PNs, cell viability was measurement by MTT (3‐(4,5‐dimethylthiazol‐2‐yl)‐2,5‐diphenyltetrazolium) tetrazolium assay. Moreover we assessed the in vitro cytotoxicity of PNs towards total lymph node cells, splenocytes and bone marrow cells obtained from naïve C57BL/6 mice. To perform this assay, 2 × 10^6^ cells were seeded in a 96‐well plate using RPMI1640 (Gibco) media supplemented with 10% FBS for 72h at 37°C under 5% constant CO_2_ flow. After the incubation period, the cell viability was assessed by reduction of MTT tetrazolium salt assay.

### 2.7 Bioassay for LPS analysis

Lipopolysaccharide (LPS) is a common endotoxin that contaminates any material, and it may be present in our PNs. To verify the impact of the LPS present in our preparation when used in vivo, a bioassay was performed to quantify the expression of CD86, a classical molecule that increases its cell surface expression after LPS treatment, in mouse model. Briefly, mice were treated intravenously (iv) with 50 µL of PBS, LPS (0.05 mg/kg) or 50 µl (4.55 × 10^8^ PNs) of Nano/SSIEFARL intravenously. Six hours after injections, the animals were euthanized under anaesthesia and the spleens were collected and red blood cells removed with lysis buffer. Cells were stained with anti‐CD11‐PerCP Cy5.5 (clone N.418) and CD86‐PE (clone GL1) conjugated antibodies. The percentage of CD11c^+^ CD86^+^ cells (activated dendritic cells) were quantified through flow cytometry.

### 2.8 Uptake of Nano/SSIEFARL by antigen presenting cells

The uptake of PNs by JAWS II dendritic cells was visualized using fluoresce microscopy (EVOS FLoid). Briefly, JAWS II cells (1 × 10^5^) were cultured in a 24‐well plate overnight. Cells were treated with empty PNs (50 µL), SSIEFARL‐Rhodamine (6 µL [200 ng/mL]) or Nano/SSIEFARL‐ Rhodamine (50 µL). After 6 h of incubation the cells were washed three with PBS and fixed with 4% paraformaldehyde. Cells were permeabilized with 0.5% Triton X‐100 in PBS and blocked with PBS 5% SFB to reduce background fluorescence. Cells were incubated with anti‐CD73 (1:2000) for 2 h followed by washing with PBS and anti‐IgG/FITC (5 µg/mL) for more 1 h and washed again. The nucleus was stained with Hoechst 3342 (1:100).

### 2.9 Quantification of cytokines by dendritic cells in vitro

The cytokines were quantified in the supernatant of JAWS II cell cultures in the presence of Nano/SSIEFARL (9.1 × 10^9^ PNs/mL), SSIEFARL (0.3 ng/mL) o empty PNs (9.1 × 10^9^ PNs/mL). All cultures were performed as previously described and the PNs were added with cells in 70% of confluence. LPS and only media were used as positive and negative controls of cytokine production, respectively. After 24 h of incubation, supernatants were collected and the secretion of IL‐1β, TNF‐α, IL‐12p70, IL‐4, IL‐6 and IL‐10 was quantified using commercial enzyme‐linked immunosorbent assay (Peprotech).

### 2.10 Histological analysis

To evaluate possible toxicity in vivo groups of female naïve C57BL6 mice (10‐12 weeks old) (*n* = 3/group) were subcutaneously injected with 1.5 × 10^10^ (50 µL), 7.5 × 10^10^(100 µL) or 15 × 10^10^(150 µL) of Nano/SSIEFARL, or 100 µL of PBS (pH 7.2). Each group received three doses on an interval of 7 days each (days 1, 7 and 14^th^). At the end of 28 days, the animals were euthanized by deepening anaesthesia and lymph nodes, liver and kidneys were collected and fixed in 10% (v/v) neutral‐buffered formalin solution. After fixation, small pieces of the organs were embedded in paraffin, sectioned at 3 µm. The fragments were mounted on a slide, fixed with xylol, stained with haematoxylin/eosin and observed under an optical microscope at 40, 100 and 400x to histopathological analysis.

### 2.11 Virus production, titration and inactivation

For infection experiments, we used HSV‐1 strain KOS (ATCC VR1493). The virus was expanded and titrated in VERO cells (ATCC CCL‐81) cultured in Dulbecco's Modified Eagle's (DMEM) supplemented with 10% SFB. To titrate the virus, we used plate assay technique, the titles were expressed as plating forming units (PFU) [29]. Virus inactivation was performed by exposure to ultraviolet C (UV‐C) radiation for 15 min [30].

### 2.12 In vitro expansion of SSIEFARL‐specific CD8 T cells

To evaluate the immunostimulatory effect of Nano/SSIEFARL on primed CD8 T lymphocytes, splenocytes from C57BL/6 mice previously infected with 5 × 10^5^PFU/mL of HSV‐1, at sixth day of infection, were re‐stimulated in vitro with Nano/SSIEFARL and the frequency of SSIEFARL‐specific CD8 T cells evaluated using a specific tetramer H‐2K (b)/SSIEFARL‐ PE and flow cytometer. MHC tetramers are fluorescent systems that can bind up to four TCRs simultaneously and are applied to identify and quantify antigen‐specific lymphocytes. Briefly, spleen cell suspensions were plated at density of 1 × 10^6^ cells per well in a 24‐well culture plate and stimulated for 72 h with IL‐2 plus one of the following treatments: medium (control), inactivated virus 5 × 10^5^ PFU/mL, SSIEFARL (0.15 ng/mL; 0.30 ng/mL or 0.60 ng/mL) or Nano/SSIEFARL (9.1 × 10^9^, 1.8 × 10^10^ or 4.5 × 10^10^PNs/mL). All experiments were carried in triplicate. At the end of the period, the medium was removed, cells centrifuged (5 min, 1500 rpm) and incubated for 20 min with Fc block (2.4G2 supernatant + 2% mouse serum, and 0.1% NaN_3_). Afterwards, the cells were marked with a specific tetramer H‐2K (b)/SSIEFARL‐PE for 30 min at 37°C and with anti‐CD8 FITC (clone 2.43) antibodies. In this experiment, only CD8 T lymphocytes that express the TCR specific for the complex MHC I: SSIEFARL (CD8^+^SSIEFARL TCR^+^) were identified and quantified. The samples were analysed using a FACSCalibur BD flow cytometer and FlowJoV10.

### 2.13 Statistical analysis

The results are expressed as mean ± standard error of the mean (SEM) from three replicates in each of the three independent experiments. The Kolmogorov‐Smirnov test was applied to verify normality. Comparisons of parameters between different experimental groups were performed by the One‐way ANOVA test followed by Bonferroni's post‐test, Differences were considered statistically significant when *p* < 0.05. All statistical tests were performed using GraphPad Prism 6.01 software.

## 3 RESULTS

### 3.1 Nanoparticle production and stability

As shown in Table 1, the addition of ethanol significantly influenced the PDI (*p* *< *0.001) and zeta potential (*p* *< *0.05) of empty PNs as well as Nano/SSIEFARL formulations but did not influence the particle diameter. On the other hand, the temperature did not cause any significant influence in any assessed parameter (*p* > 0.05) (Table 2). Based on these different protocols, we set the PNs production at 40°C and addition of ethanol into the hydrophilic phase, this combination led to the final Nano/SSIEFARL product with average particle diameter of 352 ± 22 nm, PDI0.361 ± 0.009 and negatively charged 26.30 ± 35.00. The peptide loading of Nano/SSIEFARL‐FITC was 9.7 ± 4.5 ng/ml, representing an encapsulation efficiency of 4.9 ± 2.2 % of the theoretical calculated amount. The remaining peptide was detected in the wash samples (data not shown).

**TABLE 1:** Influence of ethanol on the physicochemical parameters of PNs loaded with or without SSIEFARL. Data are expressed as mean ± SD of three replicates and are representative of three independent experiments.*p < 0.05, **p < 0.01 and ***p < 0.001. The tests were performed at room temperature

Formulation	Size (nm)			PDI			Zeta Potential (mV)		
	No EtOH	EtOH	*p* value	No EtOH	EtOH	*p* value	No EtOH	EtOH	*p* value
Empty PNs	362 (±14)	329 (±21)	0.228	0.643 (±0.010)	0.434 (±0.009)	***	−19.33 (±2.51)	−25.60 (±0.57)	*
Nano/SSIEFARL	413 (±24)	352 (±22)	0.485	0.502 (±0.004)	0.361 (±0.009)	***	−17.20 (±1.58)	−26.30 (±1.35)	*

**TABLE 2:** Influence of temperature on the physicochemical parameters of PNs produced. Data are expressed as mean ± SD of three replicates and are representative of three independent experiments.*p < 0.05, **p < 0.01 and ***p < 0.001. The tests were performed at room temperature

Formulation	Size (nm)			PDI			Zeta Potential (mV)		
	40°C	90°C	*p* value	40°C	90°C	*p* value	40°C	90°C	*p* value
Empty PNs	240 (±14)	237 (±29)	0.785	0.346 (±0.046)	0.330 (±0.010)	*0*.635	−20.33 (±2.08)	−24.00 (±4.58)	*0.127*
Nano/SSIEFARL	287 (±21)	275 (±31)	0.412	0.360 (±0.017)	0.350 (±0.034)	0.422	−17.00 (±5.56)	−20.00 (±4.58)	0.611

**FIGURE 1:**
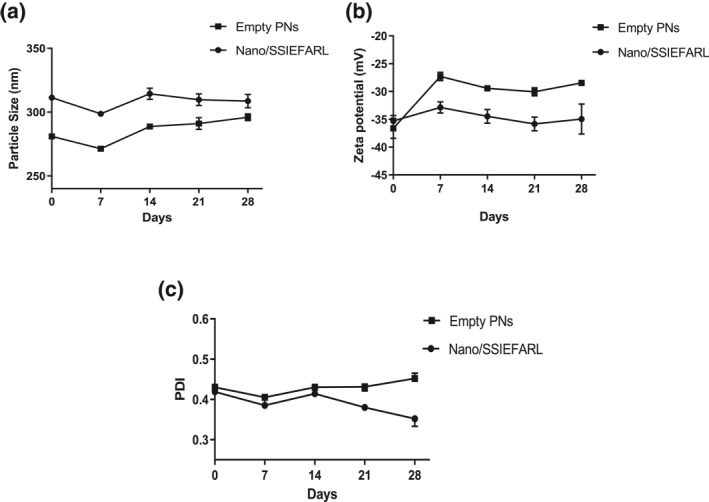
Three physicochemical parameters of empty or Nano/SSIEFARL PNs stored under refrigeration at 4ºC were assessed over 28 days (a) Particle size; (b) Zeta potential and (c) PDI

As shown in Figure 1a–c, there were no statistically significant alterations in physicochemical parameters of the empty and Nano/SSIEFARL PNs along 28 days of storage at 4°C.

### 3.2 Morphological analysis and encapsulation of SSIEFARL

**FIGURE 2:**
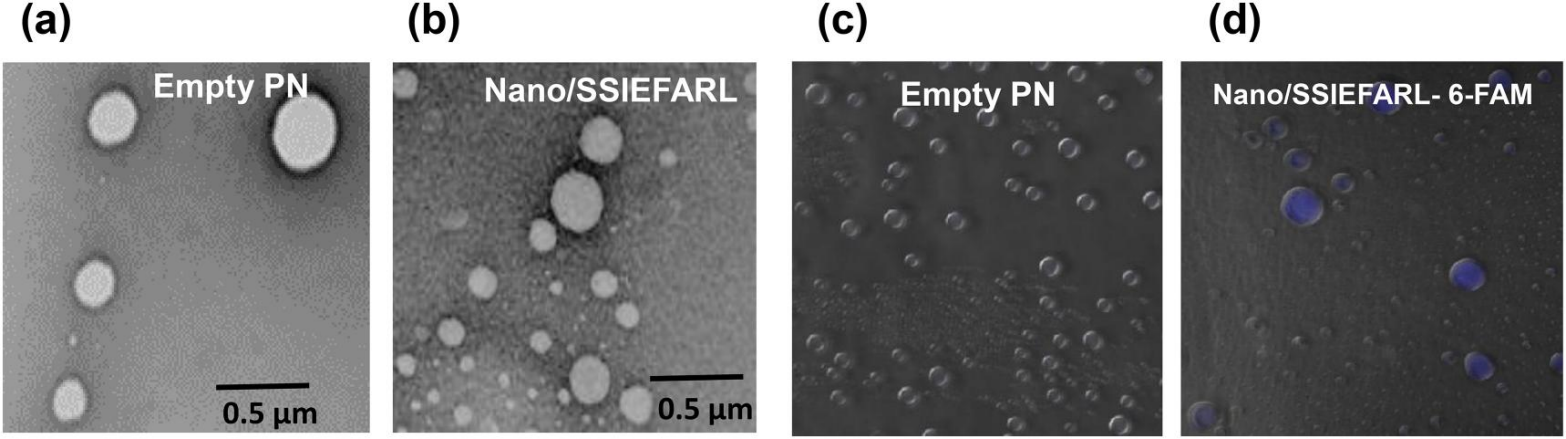
Transmission electron microscopy of PNs. (a) Empty PNs; (b) Nano/SSIEFARL; (c)Confocal laser scanning microscopy of empty PNs; (d) Nano/SSIEFARL

TEM analysis showed that PNs (empty and Nano/SSIEFARL) have a spherical shape with average diameters between 350 and 400 nm (Figure 2a,b). This observation is in agreement with the size observed by DLS. To visualize encapsulated SSIEFARL into PNs, Nano/SSIEFARL 6‐FAM PNs were evaluated by CLSM. As shown in Figure 2c empty PNs did not show any fluorescence, while Nano/SSIEFARL 6‐FAM displayed a blue fluorescence (2D), mainly inside spherical structures. It was observed that the peptide is continuous and slowly released from Nano/SSIEFARL‐FITC, reaching less than 25 % of the theoretical concentration in 24 h. Taken together, these data indicate that the PNs produced have nanotechnology adequate morphology and size, and the SSIEFARL peptide is inserted into PNs matrix while the release of the peptide to external media is controlled.

### 3.3 Viability of dendritic cells cultured with PNs and particles uptake

**FIGURE 3:**
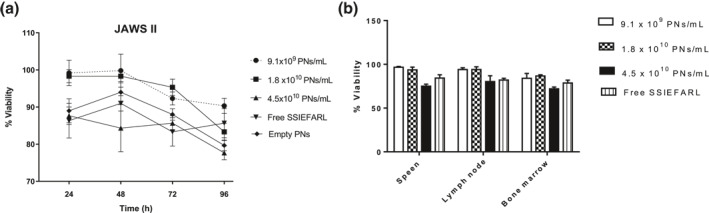
Effects of PNs on cell viability (a) Viability of JAWS II measured by MTT assay after 24, 48, 72 and 96 h of incubation. Cells were treated with 9.1 × 10^9^, 1.8 × 10^10^ or 4.5 × 10^10^ PNs/mL of Nano/SSIEFARL, free SSIEFARL or 1.8 × 10^10^ empty PNs/mL.(b) Viability of cells isolated from spleen, lymph node and bone marrow from C57Bl/6 mice and cultured for 48 h with 9.1 × 10^9^, 1.8 × 10^10^ or 4.5 × 10^10^ PNs/mL. The data from MTT assay are results from three independent experiments

The cytotoxicity of PNs against dendritic cells was evaluated by MTT assay. We used the concentrations of 9.1 × 10^9^ PNs/mL, 1.8 × 10^10^ PNs/mL or 4.5 × 10^10^ PNs/mL in all experiments. The results from MTT assay showed up to 90% of cell viability for all samples tested(*p* > 0.05) at 24 and 48 h (Figure 3a). A reduction in the cell's viability to 85% and 75% was observed at 72 and 96 h of incubation, respectively, in a culture with 4.5 × 10^10^ PNs/mL. The reduction in cell viability was significantly different (*p* < 0.05) only upto 9.1 × 10^9^ PNs/mL comparing 24 and 96 h. The cell viability observed in treatment with empty PNs (1.8 × 10^10^ PNs/mL) was equal (*p* > 0.05) as Nano/SSIEFARL at the same amount. Free SSIEFARL did not influence (*p* > 0.05) the cell viability. To complement the cytotoxicity analysis of PNs, we used MTT assay to determine the viability of primary cells isolated from spleen, lymph node and bone marrow from C57BL/6 mice after 48 h of culture in presence of Nano/SSIEFARL or free SSIEFARL. As shown in Figure 3b, again the cell viability was around 95% in any amount of Nano/SSIEFARL tested.

**FIGURE 4:**
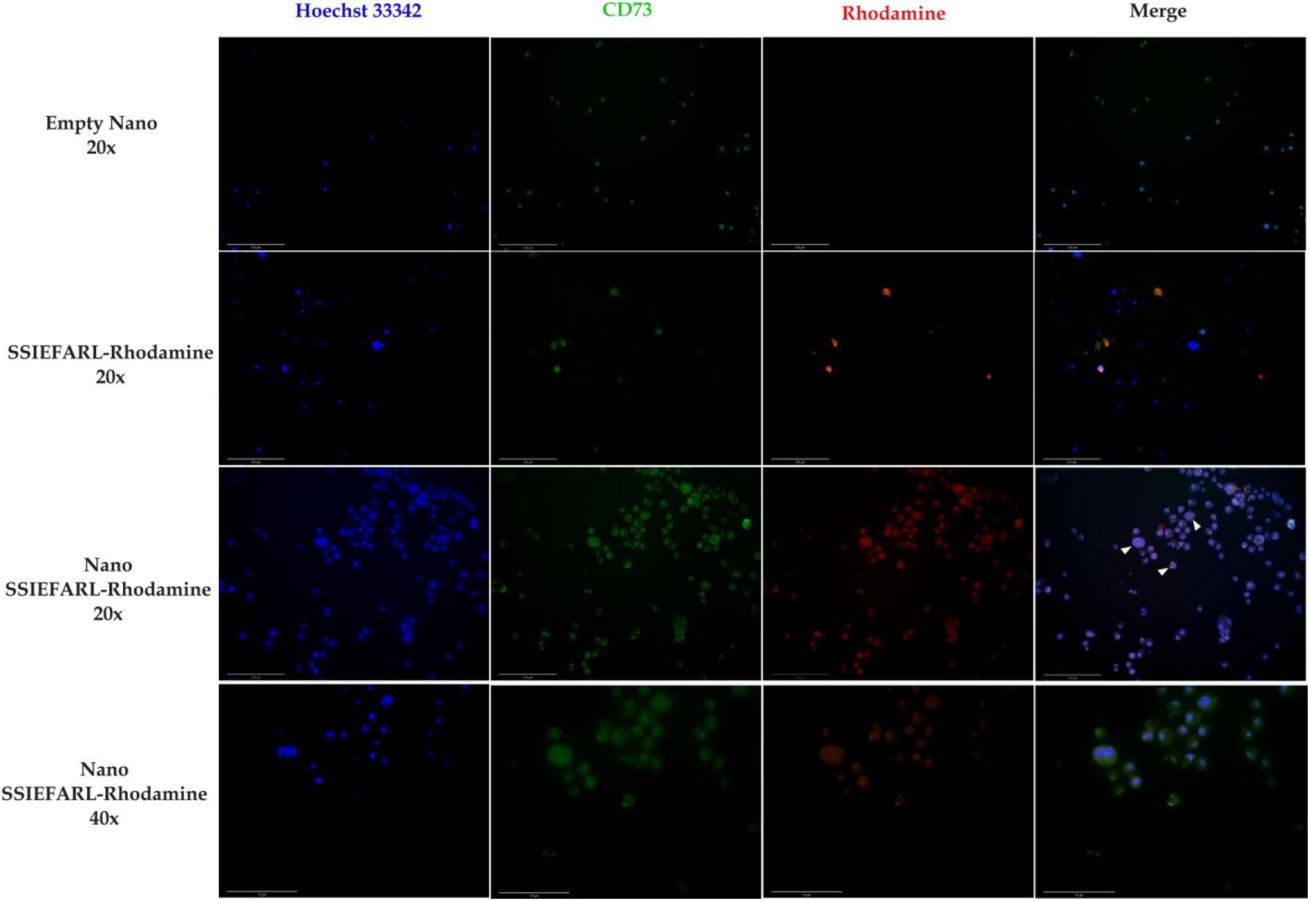
Uptake of Nano/SSIEFARL by JAWS II cells. Cells were cultured till confluence and treated with empty PNs, SSIEFARL‐rhodamine or Nano/rhodamine for 6h followed by staining with Hoechst 33,342 and anti‐CD73 FITC. The presence of Nano/SSIEFARL‐rhodamine dots is better observed on 40× marge plate

One of the major goals of this study is to produce a system that could be efficiently up taken by dendritic cells, since these cells present peptides to T lymphocytes to induce adaptive immune response. To determine if PNs are efficiently uptake by dendritic cells in vitro*,* JAWS II were cultured with empty PNs, SSIEFARL‐rhodamine or Nano/SSIEFARL‐rhodamine and subsequently evaluated by fluoresce microscopy. Figure 4 shows a table of images from JAWS II treated with empty Nano, SSIEFRAL‐rhodamine or Nano/SSIEFRAL‐rhodamine. Cells were stained for nucleus with Hoechst 33,342, and cytoplasm with anti‐CD73 FITC. The merge from cells treated with Nano/SSIEFARL shows yellow dots of rhodamine in cytoplasm. Together, these results indicate that PNs produced did not reduce the viability of JAWS II cell line and primary C57BL/6 mice cultured cells when used at a concentration ≤1.8 × 10^10 ^PNs/mL into these cultures, and Nano/SSIEFARL may be internalized by the dendritic cells.

### 3.4 Cytokine induction and cell markers

**FIGURE 5:**
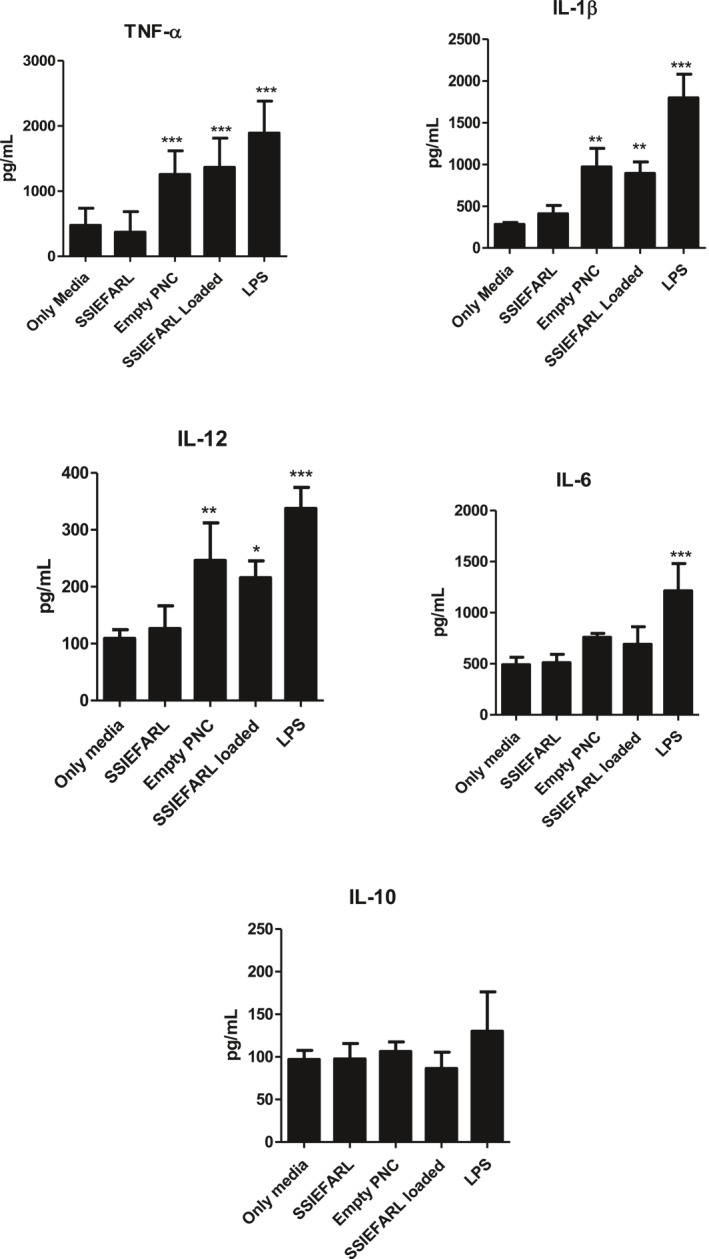
JAWS II cells were cultured in presence of Nano/SSIEFARL (1.8 × 10^10^ PNs/mL), empty PNs (1.8 × 10^10^ PNs/ml), SSIEFARL (0.3 μg/mL) or LPS (100 µg/mL) for 24 h and the supernatant was used to quantify the cytokines by ELISA. The data are mean of at least three independent experiments. Significance was calculated by One way ANOVA followed by Dunne's post‐test. *p < 0.05, **p < 0.01, ***p < 0.001 and *p < *0.0001 versus media.

We evaluated whether PNs could induce the production of cytokines by JAWS II in vitro. JAWS II cells were cultured in the presence of Nano/SSIEFARL (1.8 × 10^10^ PNs/mL), empty PNs (1.8 × 10^10^ PNs/ml), SSIEFARL (0.3 μg/ml) or LPS (100 µg/ml) for 24 h and the supernatant used to quantify the cytokines by ELISA. As depicted in Figure 5, a significantly induction of TNF‐α by JAWS II was observed in cells treated with empty PNs (1257.2 ± 360.9 pg/mL) and Nano/SSIEFARL (1365 ± 449.1 pg/mL) compared to cells cultured with media (477.3 ± 260.7 pg/mL). The same pattern of induction was observed for IL‐1β production, empty PNs treatment presented 969.7 ± 223.9 pg/mL and Nano/SSIEFARL treatment reached a mean of 891.7 ± 139.3 pg/ml, both significantly higher than treatment with culture media (282.3 ± 22.5 pg/mL). When we analysed the IL‐12 production, a significant difference was also observed in empty PNs (246.3 ± 65.9 pg/mL) and Nano/SSIEFARL (215.7 ± 29.9 pg/mL) compared to the culture media (109.0 ± 15.4 pg/mL). No significant increase in IL‐6. production was observed when dendritic cells were cultured with PNs compared to media. All cytokines evaluated displayed significant difference among positive (LPS) and negative (only media) controls and no difference between media and free SSIEFARL. There was no increase of IL‐10 by any treatment.

### 3.5 Analysis of Nano/SSIEFARL using in vivo models

**FIGURE 6:**
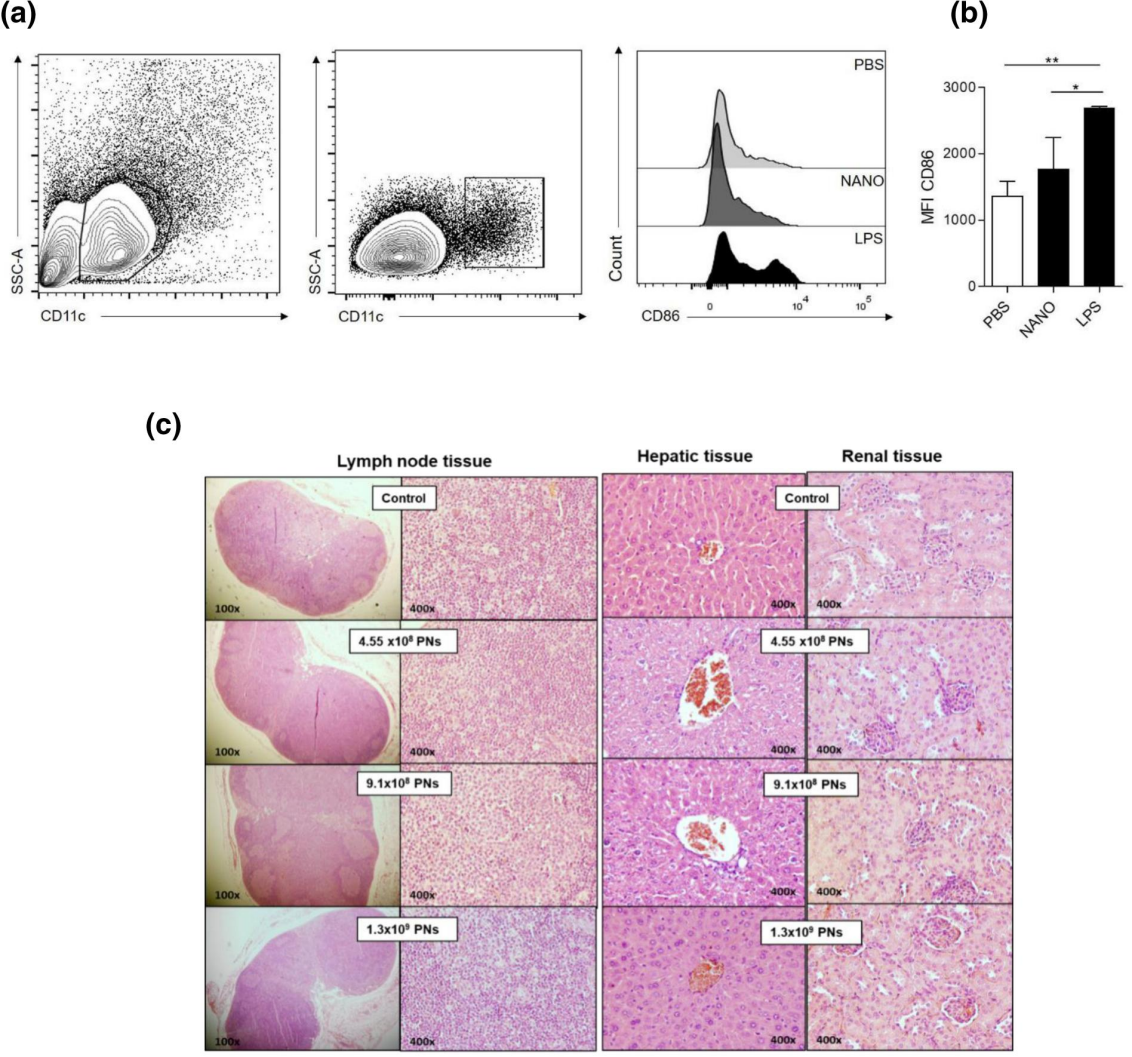
Bioassay and histological analyses: Mice were injected by retro orbital vein with 50 µL (4.55 × 10^8^ PNs) of Nano/SSIEFARL, 1 µg of LPS or 50 µL of sterile PBS and the expression of CD86 quantified in spleen dendritic cells (CD11c+). (a) Gate strategy and histogram of CD86 expression. (b) Representative graph of MFI levels of CD86 expression in treatments with PBS, PNs and LPS. The symbol * indicates *p<*0.05. (c) Histological analyses under haematoxilin/eosin stained inguinal lymph node, liver and kidneys of mice injected with S.C. in the rear thigh with 50 µL (4.55 × 10^8^ PNs), 100 µl (9.1 × 10^8^ PNs) or 150 µl (1.3 × 10^9^ PNs) of Nano/SSIEFARL, three times with 1‐week interval between each treatment. The bioassay is the result of an experiment in which three animals per group were used

Even we have used sterile materials, LPS could still present in our preparation. To determine if the LPS present in the PNs is significant to cause biological alterations we performed a bioassay to quantify the expression of CD86, a classical cell surface marker that is increased by LPS. Mice were injected by retro orbital vein with 50 µL (4.55 × 10^8^ PNs) of Nano/SSIEFARL, 1 µg of LPS or 50 µLof PBS and the expression of CD86 quantified in spleen dendritic cells. To quantify CD11c^+^ and CD86^+^ cells, we used the gate strategy presented in Figure 6a and supplementary Figure 1. First, a gate was made on total cells [side scatter (SSC) versus Forward scatter (FSC)] and the population selected was analysed for the expression of CD11c^+^ cells (dendritic cells). The expression of CD86 was quantified by level of mean fluorescence intensity (MFI) obtained in each treatment (Figure 6b). Only treatment with LPS significantly increased the expression of CD86 on splenic CD11c^+^ dendritic cells, compared to PBS. The frequency level of CD86^+^ CD11c^+^ cells in Nano/SSIEFRAL treatment was similar to the PBS group, which indicated that the amount of LPS present is not sufficient to induced dendritic cell activation.

To complement the analysis of the toxicity of PNs in vivo*,* we assessed organ damage after three SC treatment with Nano/SSIEFARL (days 1, 7 and 14). The animals showed no change in weight over three weeks (data not shown). No cellular alterations were observed in inguinal lymph node, liver and kidneys of treated mice (Figure 6c). Thus, these results suggest that Nano/SSIEFARL in those doses tested are not toxic for the animals, considering the evaluated parameters.

### 3.6 In vitro expansion of previously generated SSIEFARL‐specific CD8 T lymphocytes

**FIGURE 7:**
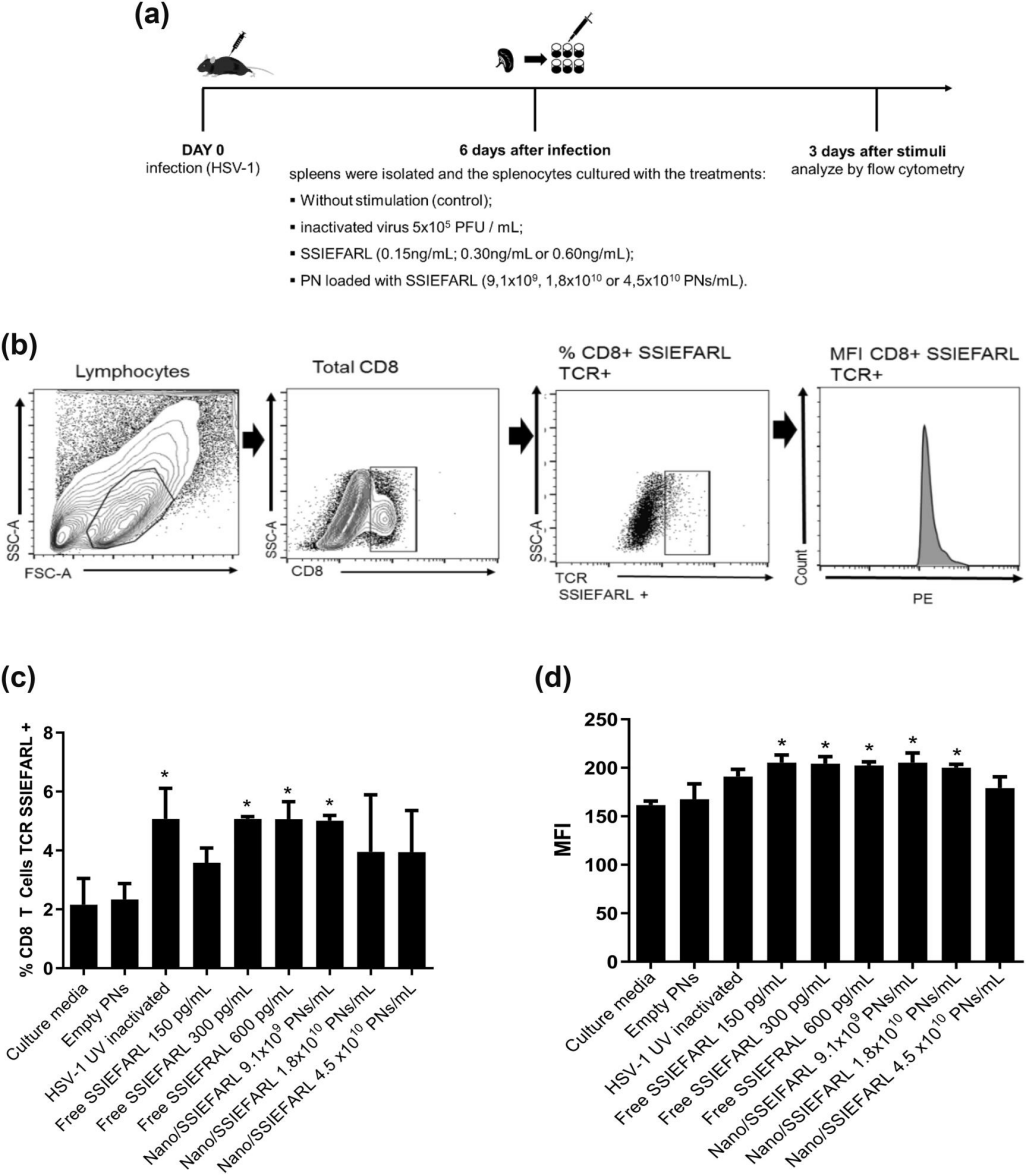
Ex vivo expansion of SSIEFARL‐specific CD8^+^ T lymphocytes (CD8+SSIEFARL TCR+). (a) The schematic representation of the experimental procedure. (b) Gate strategy to characterize the CD8 + SSIEFARL TCR + cell population inside the total splenocytes. (c) Percentage of CD8+SSIEFARL TCR + cells recovered in each treatment. (d) MFI expressed by CD8+SSIEFARL TCR + cells recovered in each treatment. Representative data from one experiment with triplicate of each treatment

As the main goal in producing Nano/SSIEFARL is its future application in preventing herpes recurrence, in which the infected host already has CD8 T cells specific for SSEIFARL, we tested if Nano/SSIEFARL could enhance the ex vivo proportion of previously induced SSIEFARL‐specific CD8^+^T lymphocytes. A scheme of the experiment design is presented in Figure 7a. The expanded cells were analysed by flow cytometry and a cell gate strategy was applied to characterize the CD8^+^ T cells with TCR specific to SSIEFARL (Figure 7b). The efficiency and specificity of the tetramer were demonstrated in cells directly isolated from the infected mice (supplementary Figure 2). The results are present in percentage of CD8^+^ SSIEFARL TCR^+^ and MFI. We observed a significant increasing in percentage of CD8^+^SSIEFARL TCR^+^ cells when the cultures were treated with UV inactivated HSV‐1, 300 pg/ml, 600 pg/ml of SSIEFARL and 9,1 × 10^9^ PNs/mL of Nano/SSIEFARL comparing to cells cultured with media (Figure 7c). When we analysed the (MFI) of the CD8^+^SSIEFARL TCR^+^ from each treatment, a significant increase was observed in treatments with UV inactivated HSV‐1, free SSIEFARL 150 pg/mL, free SSIEFARL 300 pg/mL, free SSIEFARL 600 pg/mL, 9.1 × 10^9^ PNs/mL and 1.8 × 10^10^ PNs/mLin relation to cells cultured only with media (Figure 7d). There was no significant difference between the treatments with free SSIEFARL and Nano/SSIEFARL. Taken together these results suggest that the encapsulation of SSIEFRAL did not impact the frequency of CD8^+^SSIEFARL TCR^+^ cells in vitro, however, the stimulatory capacity of the SSIEFARL is maintained when it is delivered in nanoparticulate system.

## 4 DISCUSSION

Immunotherapy is a new methodology that has been used to treat chronic degenerative diseases, cancer and viral infections [31, 32, 33]. This therapy stimulates the immunological system to respond or fight against the aggressors. When the target for immunotherapy is the adaptive response the stimulation is mediated by antibodies, cytokines or lymphocytes [34]. The immune response against HSV‐1 and HSV‐2 is orchestrated by antibodies produced by B cells, CD4^+^ and CD8^+^ T cells and cytokines such as TNF‐α, IL‐1,IL‐12 and interferons, that control the kinetics of immunological cells [35]. The recurrence of HSVs is controlled specially by CD8^+^ T lymphocytes, which after a single episode, the remaining cells will influence the next viral reactivation [36]. In this study we produced a nanostructured system to deliver SSIEFARL, a CD8 T lymphocyte immunodominant epitope of HSV‐1 and 2, with a future perspective to produce an immunotherapeutic product for mucosal application. To the best of our knowledge, this is the first report the applied nanotechnology to improve the use of SSIEFARL for immunotherapy.

The production of Nano/SSIEFARL was optimized by testing different production protocols that included forms of agitation (data not shown), addition of ethanol and different temperatures (Tables 1 and 2). The addition of ethanol improved the physicochemical properties of Nano/SSIEFARL since it reduced the particle size and PDI. This effect was also seen elsewhere and can be attributed to Marangoni effect driven by chemical potential difference among solvent phases on the emulsified system [37]. All PNs produced presented negative charge which make the particle aggregation and alterations in size and PDI difficult due to electrostatic stabilization. The physicochemical parameters evaluated did not present significant difference over 28 days at 4°C, which allowed Nano/SSIEFARL to be used in *in vitro* and in vivo experiments. A recent study with nanoencapsulated curcumin showed that PNs with less than 20 days of stability and did not compromise the in vitro and in vivo results, if used before this time [38]. The formulation with MMA was first developed to encapsulate deoxyribonucleotides [39], and here we adapted to peptide model since the two molecules present similar amphiphilic properties. Our data showed that SSIEFARL is released from PNs in a controlled manner, and approximately three‐quarters of initial concentration remains associated to PNs after 24 h. Considering the in vivo application and the necessity of uptake of SSIEFARL by DCs or macrophages, which usually takes around 3‐4h for internalization [40, 41].

We observed that Nano/SSIEFARL does not seem to be toxic for cells in vitro or tissues in vivo*,* especially in lower doses evaluated. We hypothesized that the reduction in cell viability observed in higher doses of PNs could result from cell disruption caused by deposits of PNs on extracellular matrix, which interferes with membrane functions or cell signalling [42]. Also the sequestration of nutrients from the media by PNs can be involved [43]. Theses process are related to the formation of PNs‐protein corona complex, which is a dynamic interaction between PNs and medium or cell components frequently observed in experiments with nanoparticles and depends on several factors, including the charge density [42, 44]. Corroborates with our interpretation the observation that apoptosis, which is a death mechanism mediated by intracellular signalling, was insignificant in the viability tests with PNs, the majority of cells died by necrosis (PNs, in high concentrations).The PNs produced did not present enough amounts of LPS to induce CD86 upregulation on DC in vivo, as observed in bioassay experiment Since the future application will be in mucosa, low amounts of LPS are tolerable.

The idea of nanoparticles as systems for delivery peptides has been already explored [18, 45, 46]. Quantification of cytokines induced by nanoparticles is one the most relevant and useful parameter to evaluate and predict the level of immunogenicity. Pro‐inflammatory cytokines, such as TNF‐α, IL‐1β, IL‐12 and IL‐6 can influence the promotion of effector CD8 T cells as well as the priming for different polarizations of CD4 T‐cell mediated immune response [47, 48]. Based on the levels of TNF‐α, IL‐1β and IL‐12 induced by both empty PNs and Nano/SSIEFARL, our nanoparticulate system appears to have a self‐adjuvant property. Furthermore, our data show that this self‐adjuvant property for cytokine production has inflammatory bias, since IL‐10, a classical anti‐inflammatory cytokine, was not produced in any treatment. The induction of cytokines in vitro by both empty as well as SSIEFARL‐loaded PNs found in our study is in agreement with a study conducted by Badkas et al. (2017) showing that the incubation with PNs alone induced the production of TNF‐α by macrophages in vitro [49]. The secretion of IL‐12 was also observed in dendritic cells cultured with nanoparticles without any antigen loaded or associated [50]. The biochemical dynamics present on PNs‐protein corona complex, also called ‘bio‐corona' depends of the cargo, hydrophobicity and ligands that cover the nanoparticle and these characteristics impact on the rate of uptake by the antigen presenting cells and immunological properties [51]. Antigen presenting cells, such as dendritic cells, respond to pathogen‐associated molecular patters (PAMPs) and damage‐associated molecular patterns (DAMPs) using pattern recognition receptors (PRR) [52, 53]. Once activated these PRR induces the activation of NF‐кB pathway and the production of inflammatory cytokines. Nanoparticles coated with the bio‐corona complex can act as nanomaterials associated patters (NAMPs) and induce the cytokine production as demonstrated here and by other, probably by a mechanism mediated trough the nucleotide‐binding oligomerization domain‐, leucine‐rich repeat‐, and pyrin domain‐containing 3 (NLRP3) inflammasome [54].

To evaluate the influence of SSIEFARL encapsulation on the CD8^+^ T lymphocyte frequency we used an ex vivo model with cells previously generated in HSV‐1 infected mice. The ex vivo expansion of splenocytes is an usual experiment for screening the effects of vaccine candidate in T cell proliferation [55, 56]. Although we have not seen an increase in the frequency of CD8 T lymphocytes SSIEFARL^+^ in the treatment with Nano/SSIEFARL compared to free SSIEFARL, the increase of both groups was significant when compared to the controls (only media and empty PNs). This indicates that the encapsulation did not negatively interfere with SSIEFARL's immunostimulating ability for CD8 T lymphocytes. The level of expansion was around 5%, what seems small, however, these cells are antigen experienced lymphocytes, which, in a second encounter with its antigen, rapidly proliferate in an exponential way [13, 57, 58]. It is well established that the response of CD8 T lymphocytes is dependent on CD4 T lymphocytes, a phenomenon called CD4‐help [59, 60, 61]. Although we have not investigated, it is possible that the adjuvant effect of NPs on cytokine production also influences the CD4‐help. Since we have not yet tested this system on mucosa models, we speculate that nanoencapsulation prevents the loss of SSIEFARL due to degradation and also in the mucosa, providing better results than the free form of peptide in the same concentration. The optimization of the nanoencapsulation model, prioritizing the increase in the amount of SSIEFARL encapsulated as well as the kinetics of release, and direct analysis in vivo may result in a more potent immunostimulatory response for CD8 T lymphocytes.

## 5 CONCLUSION

We showed that the SSIEFARL immunogenic epitope from HSV 1 and 2 is suitable to be delivered by the nanoparticulate system with a significant impact on CD8 T lymphocyte stimulation. This is a promising model for immunotherapy against these viruses.

## CONFLICT OF INTEREST STATEMENT

The authors state no conflict of interest.
